# Implementation Evaluation of an AI Scribing Tool in the Ambulatory Setting: Single-Site Qualitative Implementation Evaluation Study

**DOI:** 10.2196/96245

**Published:** 2026-11-03

**Authors:** Audriana Ketchersid, Melissa Jost, Stephanie Huerta, Jennifer L. Rosenthal

**Affiliations:** 1 University of California Davis, 2516 Stockton Blvd, Sacramento, CA, 95817, United States, 1 7344719; 2 University of California San Diego, La Jolla, CA, United States

**Keywords:** AI, AI scribe, electronic health record, patient care, workflow, ambulatory care

## Abstract

**Background:**

AI scribing tools to assist health care providers by generating clinical notes are rapidly spreading across health care settings. Although these tools may reduce documentation burden, successful implementation depends on the contextual factors influencing their integration into clinical practice. Implementation evaluations are needed to inform practice integration, guide future adoption, and enhance effective use of this novel technology.

**Objective:**

This study aims to explore contextual factors influencing the implementation of an AI scribe for physicians in the ambulatory setting.

**Methods:**

This study was a single-site qualitative implementation evaluation at a metropolitan university health system where the AI scribe was available across primary and specialty care clinics. The Practical, Robust Implementation and Sustainability Model (PRISM) and the Sociotechnical Systems framework guided the evaluation. Using maximum variation sampling, we recruited attending physicians with varied levels of AI scribe use. Semistructured interviews were conducted with attending physicians working in the ambulatory setting. Data collection and analysis occurred iteratively until thematic saturation was achieved. Interviews were analyzed using thematic analysis with a constant comparative method, incorporating both deductive and inductive coding. Resulting subthemes were mapped onto corresponding domains of the PRISM and Sociotechnical Systems frameworks.

**Results:**

A total of 25 physicians were interviewed, including 7 who had never used the AI scribe, 9 who used it minimally, and 9 who used it frequently. Participants represented a broad range of specialties. A total of 5 themes and 15 subthemes emerged. First, workflow integration influenced adoption. Limitations in customization were a barrier to adoption, while flexibility among users was critical to fit. Second, ease of tool usability facilitated initial adoption, whereby users tried the tool without paying close attention to training materials. However, this lack of training engagement hindered widespread sustained use due to limited knowledge of advanced functionalities. Third, attending physicians balanced trust and confidence in AI-generated documentation with mistrust and professional accountability. Many described a “trust but verify” approach. Also, some concerns about others’ inadequate review of AI-generated content weakened physician-physician trust. Fourth, attending physicians viewed the ubiquitous presence of AI scribing as inevitable, driven by organizational priorities related to productivity, cost, and burnout. Interviewees disagreed about the appropriate use of AI scribes by trainees. Finally, identical mechanisms were viewed as both benefits and drawbacks. Increased efficiency from AI documentation was perceived by some as reducing burden and cognitive load, but perceived by others as potentially intensifying workload expectations. Similarly, some thought AI scribes facilitated more patient-centered communication, while others thought it had the opposite impact.

**Conclusions:**

Implementation of AI scribes in ambulatory care is shaped by complex sociotechnical and contextual factors. Adoption is influenced by workflow alignment, trust, and preexisting beliefs about the tool’s benefits and drawbacks, illustrating how clinical context affects AI scribe implementation.

## Introduction

AI software to assist health care providers by transcribing and summarizing clinical encounters in real-time (referred to as “AI scribes” hereafter) is becoming increasingly common across health care settings [1,2]. These tools are designed to reduce documentation burden, improve provider efficiency, increase reimbursement, and enhance clinical workflows. Prior research examining AI scribe implementation has primarily evaluated clinical documentation accuracy, efficiency, clinician workload, and physician-patient interactions, demonstrating that AI scribes reduce clinician documentation workload, allowing more time for patient care interactions [3-6]. However, recognized implementation challenges include factors such as integration with the electronic health record, note formatting, and concerns about accuracy and errors [5,7,8]. Although evidence regarding AI scribe implementation and outcomes is rapidly growing [9,10], the body of literature lacks in-depth information about the organizational and sociotechnical factors that shape adoption and implementation, particularly among physicians with different levels of usage, including nonuse. Given the rapid and expansive application of AI scribes across diverse health care settings, implementation evaluations are needed to inform practice integration, guide future adoption, and enhance effective use of this evolving technology.

This paper presents the use of the Practical, Robust, Implementation and Sustainability Model (PRISM) [11] and the Sociotechnical Systems (STS) frameworks [12] to conduct an implementation evaluation of an AI scribe used to assist health care providers by transcribing and summarizing clinical encounters in real-time. PRISM is an implementation framework used to develop understanding of contextual factors that affect implementation success. PRISM encompasses the following domains: intervention (organizational perspective and patient perspective), recipients of the intervention (organizational characteristics and patient characteristics), implementation and sustainability infrastructure, and external environment [11]. PRISM uses reach, effectiveness, adoption, implementation, and maintenance (RE-AIM) [13,14] outcomes to measure implementation success. STS was specifically designed for the application of AI in health care delivery and consists of 3 domains: fit with the work system elements, fit with the temporal nature of work, and transparency and trust [12]. The aim of this study was to explore contextual factors influencing implementation of an AI scribe for physicians in the ambulatory setting.

## Methods

### Study Design and Conceptual Model

This implementation evaluation was a qualitative descriptive study using thematic analysis of physician semistructured interviews. This study was guided by the domains of the PRISM [11] and STS frameworks [12]. We selected PRISM to examine the broader organizational and implementation context. We selected STS to complement PRISM by capturing AI-specific sociotechnical factors related to workflow, fit, transparency, and trust. Together, the frameworks supported exploration of interactions among the technology, its users, the clinical setting, and the broader implementation context. Both frameworks informed development of the interview guide, deductive codes in the initial codebook, and interpretation and reporting of the findings. The frameworks were applied complementarily rather than merged into a new framework. Figure 1 depicts the complementary roles of PRISM and STS, presenting the domains of each framework, and how they informed the study. This paper follows the Standards for Reporting Implementation studies (StaRI) [15].

Figure showing the complementary roles of the 2 frameworks, PRISM and STS, and how they informed the study. The figure presents the distinct and overlapping domains of the 2 frameworks and shows how both informed development of the interview guide, deductive codes in the initial codebook, and interpretation and reporting of the findings.

**Figure 1.**
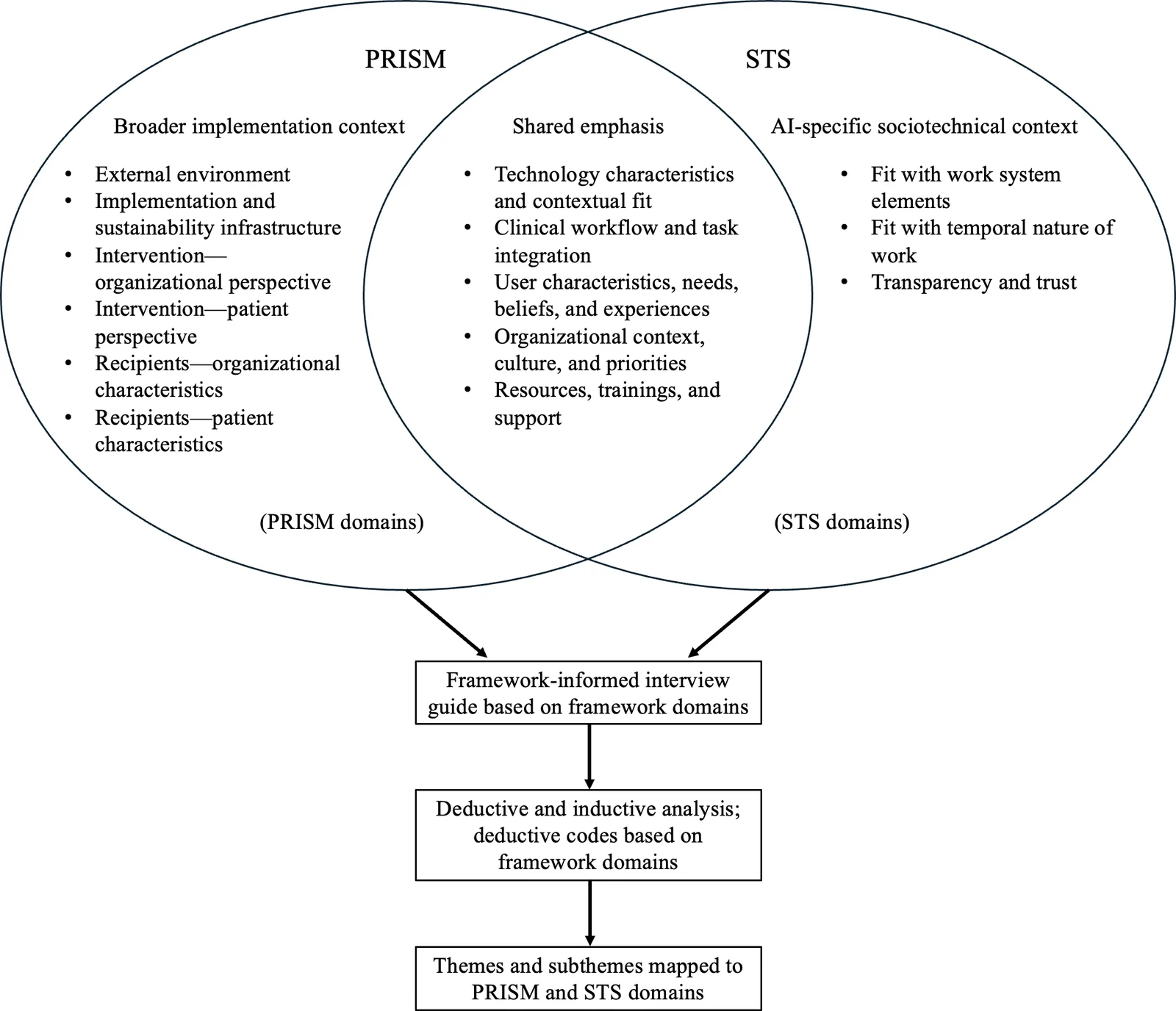
Complementary roles of Practical, Robust, Implementation and Sustainability Model and Sociotechnical Systems frameworks informing study design. PRISM: Practical, Robust, Implementation and Sustainability Model; STS: Sociotechnical Systems.

### Setting

This study took place at a metropolitan university health system with a 646-bed quaternary care hospital and ambulatory clinics comprised of 17 primary care sites and 153 specialty clinics. This health system serves as a referral center for patients across a 33-county region spanning 65,000 square miles. AI scribe services were first implemented at our health system in July 2024 with an initial pilot program with 31 physicians over 2 months. The tool was subsequently deployed to all primary care and specialty care physicians in 4 waves from October 2024 through January 2025. Additional ambulatory physicians were onboarded based on request after January 2025.

The decision to use the AI scribe was at the discretion of the physician. Physicians who elected to use the tool were required to complete an 11-minute training video prior to activation. The training covered application workflow, regulatory and compliance considerations, and best practices. The AI scribe vendor was Abridge, which is a tool that has been evaluated in prior studies [3,6]. Physicians accessed Abridge via the Epic electronic health record mobile app. An iPhone (Apple Inc.) or iPad was required for use. Where personal devices were unavailable, a limited number of shared iPads were available in clinic. The AI scribe used synchronous ambient listening to summarize conversations and structure them in real-time within the electronic health record.

### Study Population and Data Collection

We conducted one-on-one semistructured interviews with physicians between May and September 2025. Participants had to be aged 18 years and older and with English language preference. Participants were limited to attending physicians working at an ambulatory clinic affiliated with UC Davis Health where AI scribe was available for use. Eligibility was limited to attending physicians because they were the only clinicians permitted to use AI scribe at the time of study initiation.

We recruited participants via email. We used maximum variation sampling [16] to identify attending physicians with varied levels of AI scribe use; sampling was not designed to achieve balance across demographic characteristics. We strived to include participants who used AI scribe never, minimally (1 to 10 notes per week), and more frequently (exceeding 10 notes per week). No formal a priori sample size estimate was conducted. Sampling continued until we reached thematic saturation, which was defined as the point at which additional interviews generated no new substantive codes or concepts relevant to the study aim [17]. We assessed for saturation iteratively during analytic team meetings. Thematic saturation was achieved after 22 interviews; subsequent interviews reinforced and added depth to existing findings.

The interviewer (AK) used a question guide that solicited participants’ general perceptions about the AI scribe and questions aligned with the PRISM and STS domains (guide provided as Multimedia Appendix 1). Interviews were immediately followed by asking the participant to verbally self-report their characteristics, including age, gender, ethnicity, race, clinical practice location, years of experience since training, and comfort using AI scribe. Interviews were audio recorded and sent to a third-party company for transcription. Final transcripts were reviewed for accuracy by the interviewer.

### Analysis

Data were analyzed iteratively using a constant comparative approach and a combination of deductive and inductive analysis. The analysis team consisted of the interviewer plus 3 additional researchers: an attending physician and medical educator who leads digital health research initiatives for the health care system (JLR), a health information technology educator who leads clinical implementation of digital health programs for the health care system (MJ), and an undergraduate student whose scholarly focus is health technology innovations (SH). We developed an initial codebook of a priori codes related to the PRISM and STS domains.

The research team members independently conducted memo writing and coding of the initial 3 transcripts using the PRISM- and STS-based a priori codes while identifying emergent topics. The team met every other week to ensure consensus on the application of codes, refine dimensions of existing codes, add new codes as needed, develop categories, and identify theoretical direction. Disagreements among the team members were resolved through discussions to understand viewpoints, deliberate, and achieve consensus. We iteratively adapted the interview guide to explore emerging topics. We resumed independent memo writing and coding followed by team meetings; this iterative process was repeated with every 2 to 5 transcripts. Most emerging content fit within existing a priori codes, whose dimensions we refined accordingly. One inductive code (trainee curriculum) was added, and previously coded transcripts were re-reviewed for relevant content. We identified linkages and patterns between the codes to develop analytic themes and subthemes. This process continued until we achieved thematic saturation, whereby data coalesced around similar themes. We mapped each subtheme to the corresponding domains of the PRISM and STS frameworks. We used ATLAS.ti to organize and store coding and data analysis [18]. Multimedia Appendix 2 contains the final codebook.

The multidisciplinary analysis team included individuals with backgrounds in public health, medical education, medicine, and health technology. A total of 2 team members (MJ and JLR) held digital health leadership roles; one (JLR) who led digital health research and practiced as an attending physician exclusively in the inpatient setting, while the other led health information technology clinical implementation. These roles could have influenced the interpretations of AI scribe implementation, likely in a more favorable manner. To mitigate this potential bias, interviews were conducted by a nonphysician who did not hold such a leadership role and had no prior working relationship with participants. Transcripts were coded independently by members of the 4-person analysis team (AK, MJ, SH, and JLR), divergent perspectives were examined, and differences were resolved through team discussion.

### Ethical Considerations

The University of California Davis Institutional Review Board approved this study (#2307389) with a waiver for written documentation of consent to satisfy ethical reporting standards. Participants provided verbal informed consent. The third-party company deidentified the data at the time of transcription. Interviewees did not receive compensation for participation in this study.

## Results

### Overview

A total of 25 interviews were completed with physicians. The duration of each interview was approximately 30 to 45 minutes. Representation of AI scribing usage was relatively equal across groups defined by AI scribe use: never (n=7), minimally (n=9), and more frequently (n=9). Interview participant characteristics are described in Table 1. The following sections display the results of our qualitative analysis organized by theme. Overall, 5 themes and 15 subthemes emerged and are described below. Table 2 presents representative quotes supporting each subtheme, with corresponding PRISM and STS domain classification.

Characteristics of attending physicians participating in this qualitative implementation evaluation, stratified by frequency of AI scribe use. Characteristics were self-reported. Participants with prior experience using AI scribe (n=18) rated their current comfort, whereas those without prior experience (n=7) rated their anticipated comfort with using the tool.

**Table 1.** Interview participant characteristics.

	Frequent use (>10 weekly notes), n=9	Minimal use (1‐10 weekly notes), n=9	Never used, n=7	Total, n=25
Age (years), n (%)				
31‐40	3 (33)	3 (33)	1 (14)	7 (28)
41‐50	1 (11)	4 (44)	5 (71)	10 (40)
51‐60	4 (44)	1 (11)	1 (14)	6 (24)
60+	1 (11)	1 (11)	0 (0)	2 (8)
Gender, n (%)				
Male	3 (33)	8 (89)	5 (71)	16 (64)
Female	6 (67)	1 (11)	2 (29)	9 (36)
Ethnicity, n (%)				
Hispanic	4 (44)	0 (0)	0 (0)	4 (16)
Not Hispanic	5 (56)	9 (100)	7 (100)	21 (84)
Race, n (%)				
Multiracial	0 (0)	0 (0)	1 (14)	1 (4)
White	5 (56)	3 (33)	4 (57)	12 (48)
Asian	3 (33)	5 (56)	2 (29)	10 (40)
Other	1 (11)	0 (0)	0 (0)	1 (4)
Not reported	0 (0)	1 (11)	0 (0)	1 (4)
Clinical location, n (%)				
Predominantly outpatient	6 (67)	4 (44)	0 (0)	10 (40)
Predominantly inpatient	0 (0)	1 (11)	0 (0)	1 (4)
Both outpatient and inpatient (approximately equal)	3 (33)	4 (44)	7 (100)	14 (56)
Years of experience after training (years), n (%)				
0‐5	3 (33)	3 (33)	0 (0)	6 (24)
6‐10	1 (11)	3 (33)	2 (29)	6 (24)
11‐15	0 (0)	0 (0)	3 (43)	3 (12)
16‐20	1 (11)	0 (0)	1 (14)	2 (8)
21+	4 (44)	3 (33)	0 (0)	7 (28)
Not reported	0 (0)	0 (0)	1 (14)	1 (4)
Comfort level using AI scribe, n (%)				
Very uncomfortable	0 (0)	0 (0)	3 (43)	3 (12)
Slightly uncomfortable	0 (0)	1 (11)	0 (0)	1 (4)
Neutral	0 (0)	2 (22)	1 (14)	3 (12)
Slightly comfortable	0 (0)	1 (11)	1 (14)	2 (8)
Very comfortable	9 (100)	5 (56)	2 (29)	16 (64)

**Table 2.** Themes and supporting Quotes^a^.

Subtheme	Quotes	PRISM^b^ domains	STS^c^ domains
Theme 1: Workflow integration influences adoption: flexibility is critical to fit			
Rigid functionality and other limitations impede seamless integration with existing workflows	“AI doesn’t read the previous history. So if the patient has...a heart failure, a respiratory failure‚ [AI scribe] doesn’t know about what we are talking about.” -Provider 03 “My workflow is to copy forward my last note, and that includes the plan, the assessment, and the HPI^d^… if it was smart enough to figure out how I was doing things and go along with that, might have saved me some time, but I just found I had to delete and redo what it was putting into my notes. You can’t give it any instructions. You just get what you get. So it was pretty disappointing.” -Provider 11 “The AI scribe did not seem to organize the notes the way I would like it. It was more conversational but did not seem to capture what I wanted it to capture to the places where they belong… So I actually gave it back, and I said, ‘I don’t need it anymore.’ Because it definitely slowed me down.” -Provider 10	Intervention—organizational perspective	Fit with work-system elements Fit with the temporal nature of work
Physician adopters have adapted	“I write a preliminary note first, and then that includes the first line of past medical history. And then also the assessment plan from a previous note, or things of what I’m going to ask the patient… I basically do copy-pasting and small edits here and there. And then after I see the patient, I copy a note underneath this note.” -Provider 06 “I reached out to one of the Epic personnel who deals with the AI scribe, and she helped me create some dot phrases so that I have an AI template... I mean, it doesn’t include a past medical history, or a social history... so that all those things I have to build it in my note.” -Provider 15“ [I now] verbally say, ‘Oh, so you’re here to discuss your diabetes,’ and then talk about that. Then I’ll say, ‘Oh, and then next we’re going to talk about whatever.’ [AI scribe now] makes paragraphs out of that instead of one jumbled thing.” -Provider 19	Intervention—organizational perspective Recipients—organizational characteristics Implementation and sustainability infrastructure	Fit with work-system elements Fit with the temporal nature of work
Nonadopters do not want to be guinea pigs nor trainers	“I mean, I think another component would be that if it’s a well-oiled product out of beta testing and it’s working well—then sure, I’ll jump in. But... most of this stuff is not ready for the real world. And so, we’re being asked to almost provide free labor... Free feedback in a sense, to just help them improve their product.” -Provider 04 “I did hear that if you go back and edit AI scribe instead of just deleting it... it theoretically can learn from those edits... But I wasn’t getting paid to train it, and that would have just taken my own time, so I didn’t do that.” -Provider 11 “Well, OK, but what if this has improved substantially in two years, then maybe I should try it again… But hey, maybe there will be substantial improvement in a couple of years, and then it will be worth revisiting.” -Provider 14	Intervention—organizational perspective Recipients—organizational characteristics	Fit with work-system elements
Theme 2: tool usability influences training engagement: ease of use limits proficiency			
User-friendly tool causes some users to not pay attention to mandatory training	“I don’t think that it needs very much training, so I think the training wasn’t super helpful, because, you know, it was like a video I had to watch.” -Provider 02 “I think there was a video that we all were able to review... it just said, just do your visit. And even when I opened it the first time, it’s like, no need to dictate. Just do your visit. And I was just going to do it. And it was a little bit trial and error there.” -Provider 17 “I did the training. And then after that, the way I started learning how to use it was just by using it in patient encounters. And then kind of experimenting with it from there, like figuring out kind of how it would work... I think the big thing is learning how to kind of utilize the note that it generates to my liking. So yeah, that was probably what took the most amount of learning.” -Provider 25	Intervention—organizational perspective Implementation and sustainability infrastructure	Fit with work-system elements
Physicians still used tool despite limited knowledge around policies and regulations	“I think we’re not supposed to talk about psych things, but I don’t always pause it when I’m talking about psych things. That’s the only policy I can think of.” -Provider 23 “I think the only policy that I really know of is we have to ask, we have to get permission, and we have to document that we have permission. That’s all I can think of in terms of policy and regulation” - Provider 07 “I know I have to ask the patient if it’s okay to use it. But as far as other policies, I really don’t know if there are any.” -Provider 22	Implementation and sustainability infrastructure External environment	Transparency and trust
Concerns about medical-legal consequences are a barrier to use	“I feel like it’s a medical-legal problem. I do try to check. make sure that everything is correct and the way that I would write it. In medicine, we have a high litigation rate because of our patient population, so then I worry that the note’s slightly incorrect, and I had a very impersonal interaction with the patient because I was talking to the computer. I mean, how is the patient going to interpret that when it’s time to file a lawsuit?” -Provider 08 “For a lot of scenarios, I think we on the medical side, and the medical-legal side, are more hyper-concerned about this than patients are.” -Provider 20 “The notion of it delivering trustworthy things that are part of the medical-legal record with finality, my experience made me very pessimistic about that. It just didn’t work very well and made mistakes.” -Provider 14	Recipients—organizational characteristics External environment	Transparency and trust
Theme 3: trust and mistrust: balancing confidence in AI with professional accountability			
Some users trust with awareness and others trust without awareness	“I would say the trust is pretty high. I mean, me, if I’m being honest, maybe too high. I definitely try to be intentional about still reviewing, because I always look at all the assessment and plans, and I try to do my best to make sure I read over all the HPIs too and make sure that there isn’t an inaccuracy there. So I’d say it’s high. But, you know, still trust, but verify.” -Provider 07 “I would say [my trust in AI scribe is] maybe at like 65 to 70 percent… I do see the AI hallucinations sometimes where, because I’m reading carefully, I’m like, ‘That didn’t actually get said, but it kind of filled in a blank for me.’” -Provider 24 “I’m presuming that it’s understanding Pashto… I presume that there is a capacity for AI scribe to recognize other languages than English and Spanish.” -Provider 01	Intervention—organizational perspective Recipients—organizational characteristics	Transparency and trust
Mistrust impedes adoption, but trust can grow with use	“I could see maybe culturally, at least in the beginning, because people are aware that there’s such a thing as AI hallucinations, that it can be inaccurate, they might worry that it might be a little bit off.” – Provider 24 “Initially my trust level was low... I thought it would be too much like a binary thing. I just didn’t think it would be able to give me any type of background or context to anything. But that easily improved my trust when I saw that it could give me the context I need.” -Provider 17	Intervention—organizational perspective Recipients—organizational characteristics	Transparency and trust
AI scribing can weaken confidence and trust among the care team	“I really worry about—you’re starting to have a telephone effect. A med student asked the patient the question. If the AI gets it wrong, then the note goes to the faculty member who never asked that question at all—has to assume that the med student was correct in that info. And is now signing off on something that may be incorrect that the med student missed. But the attending doesn’t have any way of realizing it is an incorrect statement.” -Provider 13 “It made me feel cautious about when I see a referral from somebody who’s using AI, I’m like, I don’t actually know if it’s accurate. The experience was bad enough that it contaminates my opinion of what I’m getting from other providers that are using it.” -Provider 14 “I definitely second-guess some of it because I don’t know well everyone proofreads it. So I definitely sometimes have questions about how accurate everything is there. Although, I can say some of my colleagues typically write really short notes, so sometimes this actually gives me more information. But I definitely have some questions about when I see that it’s a pure AI scribe.” -Provider 23	Recipients—organizational characteristics	Transparency and trust Fit with work-system elements
Theme 4: AI in medicine: ubiquitous presence of AI scribing is inevitable			
Drivers of AI scribing use relate to cost and efficiency	“I’ve had discussions with people about having a human scribe, and one of the things that I have learned about fine-tuning a human scribe, requires time for training... So one of the challenges is the time it takes to train a scribe to your own specifications and to your own style.” -Provider 01 “In this day and age, we have to be more efficient. If it really does improve efficiency without costing that much more money. Rather than a personal scribe with whatever cost that is for every single person... if [AI scribe] improves efficiency, then of course it’s a positive, and it aligns with everyone’s goals from a healthcare-providing standpoint.” -Provider 04	Recipients—organizational characteristics External environment	Fit with work-system elements
Some physicians desire AI scribes to expand to other tasks	“I think that decision support tools would naturally emerge from something like AI scribe. Offering suggestions for specific medications or other considerations... having AI scribe double-check on, ‘These labs are typically collected on this frequency for this type of patient struggling with this issue, it was last done at this point. Do you want to order some labs?” -Provider 12 “Certainly I think that in the world of documentation, appeals letters, or things like that... Educational handouts, educational information—but again, still need to be proofread... You may be able to use it to also kind of chart ‘Has this patient been on this medication?’ for a quick ability to dive through existing analysis of prior charts, prior information, that sort of thing.” -Provider 13 “Routine chart tasks. I would love for AI to give me a summary of what’s in my in-basket, to suggest a reply to a patient’s message, or for me just to say, ‘Yes,’ and it goes through and clicks the buttons that I need to click to send a routine prescription refill, for example... Scheduling stuff with patients… Writing letters on patients’ behalf, filling out disability forms... Prior authorization. Fight the insurance companies’ AIs with our AI… Scan the chart and tell me where my care is meeting or failing to meet the guidelines for care.” -Provider 11	Intervention—organizational perspective External environment	Fit with work-system elements
Potential to enhance or hinder trainee learning	“… help [medical students] see the algorithm or how some things cascade. And that’s what I’ve seen with AI… gets a student thinking, okay, yeah, that’s what we’re worried about... That is really big because we didn’t have those internal prompts in medical school... So it really could be such an advantage for them to kind of just get everything in an organized manner.“ -Provider 17 .”..it helps to save them time, and it sort of provides more time for [attendings] to discuss the patient in greater length, then that’s one benefit, potentially, of extending this to trainees.” -Provider 15“Part of how medical students learn about medical decision making is by writing notes. For early learners, I think writing notes is an important part of their learning because it’s their opportunity to kind of pull data together and then put it—the logic of learning how to make clinical decisions‚ that is part of that process for early learners.” -Provider 02	Recipients—organizational characteristics	Fit with work-system elements
Theme 5: Divergent Perceptions: The Same Mechanisms are Viewed as Both Benefits and Drawbacks			
AI scribing allows physicians to see more patients	“If I didn’t have AI scribe, they would probably not schedule as many patients for me, and so I won’t make as many RVUs.” -Provider 06 “…I think we’re also trying to be a little bit more cost efficient. So if the scribe allows you to see more people more efficiently, as well as maybe save some funding, then great. But I think we’re doing less work in the clinic by not having to type as much, but I don’t think people are actually seeing more patients to compensate for the cost of the scribe program.” – Provider 09 “But I think the other concern I would have is that, are physicians going to be required to see more patients now that you have an AI scribe? It’s already hard enough to see as many as we do because we’re a specialty clinic. And everyone says, well, AI just writes your notes for you.” -Provider 08	Intervention—organizational perspective Recipients—organizational characteristics	Fit with the temporal nature of work
AI scribing impacts provider-patient communication	“I think that some of it could become very impersonal. With my... human scribes, I don’t really talk out loud much... I think [AI scribe] could make the clinical interaction more clunky... When you’re talking out loud to a computer, it feels like maybe that could be problematic... it’s kind of awkward to talk out loud to a machine while you’re in with the patient.” -Provider 08 “I would have to use my cell phone, and I try to not have my cell phone in clinic rooms when I’m with patients both in terms of actual distraction and perceived distraction; like, patients feeling like I’m on my phone. And it runs down battery... I kind of thought it would interfere with my clinic workflow and my relationship building with families in the room.” – Provider 20 “I think that helps me feel like I can communicate better with patients, because I don’t have to type and talk at the same time... oftentimes, I’m still in the EMR looking at things as they’re talking to me… I can focus more on talking to them and less on typing, which is really nice.” -Provider 02	Intervention—organizational perspectiveIntervention—patient perspective	Fit with work-system elements
AI scribing reduces cognitive load and time spent on documentation	“The process of having to think through the note actually changes and solidifies your understanding of what’s going on. If we replace that process, it helps efficiency, but what is it changing about our learning, our understanding? Sometimes to this day, as I’m writing the note on someone, I have a new insight about what might be going on with them that didn’t come to me in the moment when I was in the room.” -Provider 20 “I think the application that’s the most useful is around the note writing, because it actually offloads my work… work that I would do in addition to my day… at night after I put my kid to sleep, I would be writing my notes, and so that has been really helpful because I no longer have to generate the notes myself… I can just edit them and add whatever additional pieces I have.” -Provider 02 “…when you take away the fact of us having to multitask orders and actually writing down what our patients are telling us for medical documentation, that right there is reducing multitasking… We’re going to do our task better, which is to take care of our patients when we don’t have to document… the provider is actually able to be more engaged and more present with regards to the actual the visit, versus wondering about or just being stressed out or having the additional mental load of I need to document everything that is being addressed or said.” -Provider 07	Intervention—organizational perspective Recipients—organizational characteristics	Fit with work-system elements Fit with the temporal nature of work

a Five themes, 15 subthemes, and representative quotes from interviews with attending physician interviewees. Each subtheme was mapped onto Practical, Robust, Implementation and Sustainability Model and Sociotechnical Systems domains.

b PRISM: Practical, Robust Implementation and Sustainability Model.

c STS: Sociotechnical Systems.

d HPI: history of present illness.

### Theme 1: Workflow Integration Influences Adoption: Flexibility Is Critical to Fit

A common issue raised by most participants was that AI scribe inadequately integrated into their existing workflows. Several emphasized that the tool’s output misaligned with their established templates. They highlighted that using AI scribe required them to make significant edits to fit their preferences. Many nonusers said their workflow for writing notes was already efficient and AI scribe would provide no benefit. It was also noted that AI scribe did not include the patient’s past medical history, and since a patient’s past medical history is often not discussed out loud during the clinic visit, the note omitted this critical information. Physicians ubiquitously shared how AI scribe was not helpful in documenting the physical exam, as that would require extensive audible explanation and would be cumbersome and potentially uncomfortable for the patient. Nonuser and low-users alike expressed concern over AI scribe generating notes that were bloated and lengthy.

In contrast, several physicians shared how they had adapted their workflows to align with the tool’s functionality. These adopters described developing workarounds and hybrid documentation strategies to generate notes. For example, a few physicians explained how they changed the way they spoke, such as saying the past medical history out loud to the AI scribe before walking into the clinic room. One physician adapted their in-room communication style, using verbal signposting (“now we’re going to talk about…”) to help the tool segment note content to result in a more organized note. Many others tailored their workflows using note templates and dot phrases to compensate for sections not populated by, or poorly generated by the AI scribe.

Nonusers frequently expressed concern and resentment about being early or experimental users of the AI scribe. They frequently framed this early implementation as asking physicians to burden the task of beta testing. Some shared reluctance to adopt a tool not yet ready for real-world use; others rejected the notion to use the tool in its current state. A few perceived use of the tool to be uncompensated labor to improve or train the product. Several of these individuals described openness to revisiting adoption later, once its performance improved substantially.

### Theme 2: Tool Usability Influences Training Engagement: Ease of Use Limits Proficiency

Most physician adopters described AI scribe as being user-friendly and straightforward. Many highlighted that the mandatory training video felt unnecessary. Some shared the video added little value; they learned more by hands-on “trial and error.” Some could not recall specific content from the video; others did not even recall watching it. While retainment of training seemed limited, physicians still used the AI scribe because of its ease of use and the ability to learn in real time. Of note, video content included details and tips about the tool that would have addressed or mitigated many of the expressed issues with the tool. Lack of attention to details in the training materials not only impacted usability but also influenced physicians’ awareness about policies and regulations related to AI scribe. Despite stating they had limited knowledge on policies and regulations, these same individuals still used the program.

Medical-legal risk emerged as a barrier to use for some physicians. A few shared concerns that inaccuracies in a signed note could create liability. One physician noted that these medical-legal risks could be enhanced from patients perceiving the encounter to be “impersonal” because the physician had to speak into their device to use the tool. At the same time, one participant shared that physicians’ worries about medical-legal consequences likely exceed patients’ concerns. Regardless, physicians responded to their medical-legal concerns by being very vigilant about proofreading and editing practices. This perceived need for vigilant review of notes, however, limited adoption.

### Theme 3: Trust and Mistrust: Balancing Confidence in AI With Professional Accountability

Physicians shared varying levels of trust in using AI scribe, and AI in general, ranging from complete skepticism to near-complete trust. Most articulated a “trust but verify” approach, explaining that AI scribe captured details of the clinical encounter thoroughly and accurately, but that it was the physician’s responsibility to review, detect, and correct inaccuracies or “hallucinations.” While most users spoke about the importance of professional accountability, others acknowledged that their trust level was likely higher than it should be. Furthermore, a few physicians demonstrated implicit trust. For example, one physician assumed the tool accurately captured encounters conducted in languages other than English, including languages they did not speak and thus could not independently verify.

Several participants described mistrust as a barrier to adoption. They shared how the potential for inaccurate documentation necessitated time-intensive review of the generated notes, which they perceived diminished potential efficiency gains. One physician, however, noted that their trust increased over time through use and experience with the tool, resulting in a deeper understanding of the tool’s limitations and the implementation of strategies to mitigate them, such as hybrid documentation approaches.

Beyond individual trust, a few physicians expressed how AI scribe led to their mistrust in other care team members. Multiple physicians recounted examples of reading notes from other physicians that had inaccuracies that were overlooked and not corrected. One physician shared an example of receiving a note with recommendations in the assessment and plan that were outside the signing physician’s scope of practice. Specifically, a primary care physician included recommendations that would typically require surgical subspecialist evaluation and management, suggesting they were AI-generated and not appropriately edited. Similarly, another expressed worries that AI scribes used by medical students may increase the risk of inaccurate information, as supervising physicians might assume the information was accurately obtained when signing the student’s AI-generated note. Some physicians raised concerns about a “telephone effect,” whereby information inaccurately transcribed could be propagated in the electronic health record. These concerns intersect with the perceived medical-legal risk described in Theme 2.

### Theme 4: AI in Medicine: Ubiquitous Presence of AI Scribing Is Inevitable

All interviewees expressed that use of AI in health care will become ubiquitous. Multiple nonadopters stated that they will likely have to eventually use AI for clinical care, including AI scribes. The perceived main driver of widespread integration was efficiency in health care delivery. Many framed this topic of efficiency as an organizational pressure to improve productivity and reduce cost. Some contrasted AI scribes with human scribes, sharing that training human scribes required substantial time and effort and may therefore be more costly compared with AI-supported documentation. While most spoke about AI scribe improving efficiency by reducing documentation burden, a few physicians questioned its value. Those with this divergent view explained that AI scribe slowed their workflow and reduced their efficiency. Others questioned whether efficiency gains offset high licensing cost. Aside from cost, another driver that was less commonly raised related to AI scribe reducing after-hours documentation, which was perceived to align with health system goals to address burnout.

Several physicians indicated a desire for AI scribe to expand its capabilities into other clinical workflows. Expressed interests included AI-assisted patient scheduling, medication refills, interpretation of laboratory and exam results, insurance authorizations, and responses to patient portal messages. This desire for expansion of AI applications beyond scribing was expressed both by AI scribe adopters and nonadopters.

However, physicians expressed mixed views regarding expanding AI scribe use to trainees. Some thought trainees should not be exposed to these tools, most commonly citing the value of writing clinical notes as a critical experiential learning activity that supports development of clinical reasoning skills. While most interviewees believed AI scribes would diminish learning opportunities, there was great variability in how these concerns applied across training levels. In general, apprehension lessened as the trainee stage advanced, yet the threshold for introducing these tools ranged widely.

In contrast, a minority supported early exposure. These individuals thought it was important for medical students to become familiar with AI scribes early in their education due to the inevitable rising presence of AI in health care. A few physicians described potential advantages, suggesting that AI-generated notes could show trainees how to organize written information, be a source of clinical knowledge and clinical reasoning, and allow more time for teaching by reducing note-writing burden during encounters.

### Theme 5: Divergent Perceptions: The Same Mechanisms Are Viewed as Both Benefits and Drawbacks

Physicians described several mechanisms through which AI scribe was simultaneously perceived as both advantageous and disadvantageous. Productivity was one of the most discussed topics. Some high users perceived AI scribe as advantageous because it reduced time spent writing notes, allowing them to see more patients and thus increase revenue generation. However, a few physicians worried that improved efficiency would lead to pressure to see more patients within the same timeframe, resulting in further burnout. This concern was further amplified by the perception that integration of AI scribes into clinical care is inevitable, as described in Theme 4. This difference in framing—increasing capacity versus intensifying workload—influenced physician adoption. As noted previously, others questioned or disagreed with the idea that AI scribe improved efficiency, noting the need to do extensive edits to AI-generated notes.

Physicians also had contrasting perceptions about the impacts of AI scribe use on patient-provider communication. Some expressed how the tool allowed them to be more attentive and make more eye contact during patient encounters. Specifically, they noted how standard encounters required them to constantly look at the computer screen, whereas AI scribe removed that need to continuously type. Others reported improved conversational flow and more time spent in the room as a result of AI scribe. Conversely, other physicians shared concerns that speaking aloud to a device in front of the patient could be perceived as impersonal or distracting. One physician noted that using AI scribe required them to speak differently, causing the clinical interactions to be clunky and less patient-centered.

Finally, physicians shared contrasting ways in which AI scribe impacted their cognitive load during clinical encounters. Many highlighted a positive impact, sharing how editing the AI-generated note required less cognitive effort than independently composing a note. Some physicians noted that the tool reduced the need to remember details or type information in real time, which made their experience in the patient room feel mentally less taxing or stressful. However, a few nonusers commented on the value of note-writing, especially the care plan (ie, assessment and plan), sharing how that process helped them to reflect and synthesize information. They raised concerns that reliance on AI could negatively impact how physicians process clinical information, ultimately worsening the quality of patient care. This concept overlaps with the concern that emerged about trainee learning described in Theme 4.

## Discussion

### Principal Findings

Our qualitative implementation evaluation of an AI scribe for physicians in the ambulatory setting identified multilevel contextual factors influencing successful implementation, including factors not previously reported. The 5 themes that emerged underscore the complex interplay between workflow integration, tool usability, trust in AI, and provider- and system-level drivers. Using PRISM and STS frameworks, our findings increased understanding of multilevel influences that can be leveraged or addressed to optimize outcomes of interest. As highlighted in this study, what defines a relevant and meaningful outcome may differ across users, particularly with respect to efficiency, which was variably experienced as increased capacity to see more patients—viewed as a benefit by some and a downside by others.

### Comparison With Prior Work

Prior qualitative research has examined AI scribes in the ambulatory setting, including an implementation evaluation guided by RE-AIM/PRISM [7] and a qualitative content analysis characterizing edits to AI-generated notes [19]. Findings from these studies align with our current findings, including facilitators and barriers around topics of AI-generated note construction, accuracy, workload, and physician-patient interactions. Other quantitative and qualitative studies have similarly identified workflow integration, usability, documentation quality, and trust as influences on AI scribe adoption and use [9,10,20,21]. While these prior studies have characterized physician perceptions and experiences with AI scribe, including note quality and physician edits to AI-generated documentation, our study extends the literature by shifting the focus from individual user experiences to the broader organizational and sociotechnical context in which AI scribe implementation occurs.

Our study contributed new knowledge by combining an implementation and sociotechnical approach to explore how physician experiences map to key PRISM domains—particularly organizational characteristics—and STS domains—particularly transparency and trust. While Shah et al [7] similarly applied PRISM, their sample disproportionately represented high users and did not incorporate the STS framework. By more evenly sampling physicians across usage levels, including nonuse, our study captured a broader range of experiences and provided additional insight into contextual factors impeding adoption and implementation. Beyond individual user experiences, our findings highlighted the interplay between perceived health system priorities and physician experiences, beliefs, and values during implementation. Our findings suggest that the perceived organizational emphasis on productivity and cost not only shapes AI scribe implementation but also shapes how physicians interpret external pressures to adopt these tools, contributing to a sense of inevitability that their individual agency in AI scribe adoption decisions may decline over time. Within this context, reluctance to serve as early adopters underscores a misalignment between organizational momentum for implementation and physician readiness, with some physicians explicitly resisting the perceived role as “beta testers” responsible for using a suboptimal product to contribute data to refine the technology. This resistance appeared to be driven, in part, by skepticism about whether health system motivations to expand AI scribe use were truly aligned with physician well-being. Although the tool is framed as a strategy to reduce documentation burden and thus burnout, physicians expressed mistrust in the downstream outcomes, wondering if efficiency gains would instead be leveraged to increase patient volume—a mechanism that could worsen burnout.

Trust again emerged as a construct reflecting both physicians’ perceptions of the technology itself and concerns about other physicians’ overconfidence in AI-generated documentation. Publications have previously described the influence of trust on adoption of AI scribes, noting that trust functions both as a barrier to adoption when lacking and as a facilitator of adoption when present [5,10]. Research examining the mechanisms by which trust influences implementation and impact, however, is limited. Regarding implementation, one qualitative study presented findings on clinician perceptions of AI scribing implementation, describing a concept of “safe with caution” to emphasize the importance of critically reviewing AI outputs rather than accepting them blindly [22]. This concept is very similar to the “trust but verify” approach that emerged in our study, which balanced perceived efficiency benefits with professional accountability. Participants shared that without this accountability—without verification of the AI-generated documentation—notes sometimes contained inaccurate information or content outside the physicians’ scope of practice. This over-trust without verification consequently impacted physicians’ trust in other care team members. Prior studies have reported AI scribing errors, including omissions, additions, and incorrect facts [8,9,23]. Our findings additionally raise the possibility that AI-generated documentation may include content outside a clinician’s scope of practice.

Prior research has identified workflow integration as a key determinant of AI scribe adoption; our findings extend this work by suggesting that integration is not simply about alignment with existing workflows, but rather about physician adaptability and flexibility in modifying those workflows. Physicians with high adoption described actively adapting their documentation templates to integrate with the tool’s output. A commonly used approach was development of hybrid documentation strategies that combined AI-generated and manually authored content. This approach allowed physicians to leverage the efficiency of automated documentation while maintaining control over elements of the note, including clinically relevant details, accuracy, and care plans within their scope of practice. In contrast, some were unwilling to modify established workflows, and others appeared unaware of alternative approaches. Thus, dissemination of successful use strategies (eg, stating out loud the patient’s past medical history, pausing use of the tool during the physical exam, or verbal signposting) may represent a modifiable implementation target with potentially high impact. These findings align with prior research, which has identified AI scribing adoption barriers to include mismatches between clinicians’ documentation styles and the tools’ inflexible formatting and lack of customization [21]. While the body of literature on implementation of AI scribes in health care is rapidly growing [5], clinicians and health care systems lack effective strategies to overcome these workflow integration barriers. Our study helps address this gap by identifying specific adaptive strategies used by physicians.

Our study findings also extend prior research by providing a deeper understanding around the tensions between perceived benefits and risks of AI scribes around the physician-patient relationship. Consistent with our findings, prior research suggests AI scribes result in physicians and allied health professionals spending more time interacting with the patient and less time on the computer [24], improving the therapeutic alliance with more eye contact, attention, and engagement [20]. However, also consistent with our findings, concerns remain about unintended harms of eroding patient trust and loss of empathy [25]. Our study builds on this literature by elucidating perceived mechanisms underlying these perceptions. Physicians in our study described how AI scribe shaped their behaviors, such as “clunky” speech or using their smartphone in the patient room, which in turn influenced how natural or patient-centered the interaction felt. These findings suggest that how AI scribes influence the physician-patient relationship is possibly mediated not only by time allocation, but also by altered communication dynamics. As such, optimizing implementation of AI scribes requires attention to these interactional mechanisms.

### Limitations

This study is not without limitations. First, it was conducted at a single university-based health system, which may limit generalizability to other settings. Additionally, because AI scribe products vary in workflow integration, output, and accuracy, findings from our study examining Abridge may not be transferable to other AI scribe products. We sought to capture varied perspectives through maximum variation sampling targeting AI scribe use; additional research is needed that examines implementation across diverse healthcare settings, including community-based practices. Second, maximum variation sampling focused on AI scribe utilization rather than demographic characteristics; gender and ethnicity were unevenly distributed across utilization groups. These imbalances may have influenced our findings. Future research should examine associations between demographic characteristics and AI scribe use and experiences. Third, because trainees currently do not use AI scribe within our health system, interview participants were limited to attending physicians. Perspectives of trainees and other clinical staff were not included. Consequently, findings regarding trainee use reflect attending physicians’ perceptions rather than direct experiences. As AI scribe use expands to trainees, future implementation evaluations among this population are needed. Fourth, member checking was not conducted after the transcripts were synthesized. Fifth, the self-reported experiences may be subject to recall or social desirability bias. However, the use of thematic saturation and maximum variation sampling for AI scribe utilization levels strengthened the trustworthiness of our findings. Finally, although sampling continued until thematic saturation was achieved, less common perspectives may have been missed.

### Conclusions

In summary, implementation of AI scribes in ambulatory care is shaped by a complex set of sociotechnical and contextual factors. Physicians’ adoption of AI scribe was influenced by workflow alignment, trust, and preexisting beliefs about intentions and downstream consequences. Physicians varied in whether they perceived the tool’s effects on efficiency, workload, and patient-centered communication to be beneficial or detrimental. These findings illustrate how clinical context affects the implementation of AI scribe innovations.

## Supplementary material

10.2196/96245 Multimedia Appendix 1 Semistructured interview guide on physicians’ experiences with and perspectives on AI scribe use, with topics mapped to Practical, Robust, Implementation and Sustainability Model and Sociotechnical Systems frameworks..

10.2196/96245 Multimedia Appendix 2 Final qualitative codebook with code definitions and guidance on when to apply each code. It includes deductive codes from these frameworks: Sociotechnical Systems; Practical, Robust Implementation and Sustainability Model; and Reach, Effectiveness, Adoption, Implementation, and Maintenance.

## Abbreviations

PRISM: Practical, Robust, Implementation and Sustainability Model
RE-AIM: Reach, Effectiveness, Adoption, Implementation, Maintenance
StaRI: Standards for Reporting Implementation studies
STS: sociotechnical systems
