# Supplementary material for: Implementation Evaluation of an AI Scribing Tool in the Ambulatory Setting: Single-Site Qualitative Implementation Evaluation Study
Source: JMIR Med Inform. 2026 Nov 3;14:e96245. doi: 10.2196/96245 (PMC13730405; doi:10.2196/96245)
Supplement: Supplementary file 1 [file medinform-v14-e96245-s001.docx]

**Multimedia Appendix 1:** Interview Guide

| **Topic** | **Example Probing Question** |
| --- | --- |
| **General Experience**  with AI in healthcare  with AI scribe in healthcare | - As a physician, how, if at all, have you used AI in healthcare?   - Please tell me more about your overall experience with that. - What, if any, AI tools are other physicians using that you intentionally do not use for healthcare purposes?   - Please tell me the reasons or circumstances for not using it. - Tell me about your overall experience with AI scribe. |
| **Perspectives of AI Scribe**  Organizational/provider perspective ^a^  Patient perspective ^a^  Fit with work system elements ^b^  Fit with temporal nature of work ^b^  Transparency and trust ^b^ | - What, if any, is the utility of AI scribe?   - Can you tell me about a time when AI scribe was really beneficial or useful?   - Can you tell me about a time when AI scribe was harmful, unhelpful, or useless? - What, if any, evidence exists regarding the benefits of using AI scribe on improving outcomes? - How, if at all, does AI scribe integrate with Epic, MyChart, or other technologies/tools? - How, if at all, does AI scribe affect your tasks and responsibilities? ^c^ - How, if at all, does AI scribe integrate with your clinic’s workflows/procedures?   - What about with your personal workflows/tasks? - Tell me about your trust (or lack of trust) in AI scribe?   - What would you need to enhance your confident or trust in using AI scribe? ^c^ - How, if at all, do you think AI scribe is impacting patients? *(probe for positive and negative impacts)* - How does AI scribe compare with human scribes? ^c^ |
| **External Environment** ^a^ | - How, if at all, do policies affect your AI scribe use? *(consider: legal, billing, regulations)* - What, if any, financial or administrative incentives exist for using AI scribe?   - What about financial or administrative barriers? - Tell me about your thoughts around AI scribe use among medical trainees. ^c^   - What key considerations should inform the development of an AI scribe curriculum for trainees? ^c^ |
| **Implementation and Sustainability Infrastructure** ^a^ | - How often do you use AI scribe?   - Has that changed over time? Why? - What, if any, AI scribe training/resources has been most helpful? Least helpful? - If you could make any changes to AI scribe itself or how it’s used, what would those changes be? Why? - What resources or changes would you need to increase your use of AI scribe? - What would encourage/support your long-term sustainability of AI scribe? - How, if at all, do you use AI scribe differently than others (How do you use it to meet your needs)? ^c^ |
| **Recipients**  Organizational characteristics ^a^  Patient characteristics ^a^ | - How, if at all, does AI scribe align with UCDH’s goals? Your goals? - How receptive have providers in your clinic been to use AI scribe? Why? - What is an ideal type of patient or clinical encounter that is a great fit for using AI scribe and why?   - What about a type of patient or clinical encounter that is a bad fit and why? - *If they’ve used AI scribe:* Can you think of a time when you used AI scribe where it worked really well? Walk me through that experience. - *If they’ve used AI scribe:* Can you think of a time when you used AI scribe where it worked really poorly or there was harm? Walk me through that experience. |
| **Closing** | - Is there something that you might not have thought about before that occurred to you during this interview? - Is there anything else you think I should know to understand AI scribe better? |

AI – artificial intelligence. UCDH – University of California Davis Health.

^a^ PRISM (Practical, Robust, Implementation and Sustainability Model) domain

^b^ Sociotechnical Systems framework domain

^c^ Question added during the iterative data collection and analysis process to explore an emerging topic
