# Supplementary material for: Implementation Evaluation of an AI Scribing Tool in the Ambulatory Setting: Single-Site Qualitative Implementation Evaluation Study
Source: JMIR Med Inform. 2026 Nov 3;14:e96245. doi: 10.2196/96245 (PMC13730405; doi:10.2196/96245)
Supplement: Supplementary file 2 [file medinform-v14-e96245-s002.docx]

**Multimedia Appendix 2:** Final Codebook

| **Code** | **Definition; When to use** |
| --- | --- |
| Workflow ^a^ | - Comments on time-bound work activities - How AI affects workflow or time spent |
| Trust ^a^ | - Comments on trust or mistrust with AI (for themself or others) |
| Infrastructure ^a^ | - Comments on non-time bound work elements - Examples: AI has effects on alignment with having residents, physical space, staff training, spoken language other than English, IT Support |
| External Environment ^b^ | - How policies, financial/administrative incentives and barriers influence AI use or opinions about AI |
| Patient Perspective ^b^ | - Impacts on interactions, communication, time spent, care outcomes, care quality with patients |
| Organizational Perspective ^b^ | - Utility of AI scribe - Beneficial or Harmful examples - Evidence on benefits of AI scribe improving outcomes - Comparisons to (or experiences with) human scribes |
| Sustainability Infrastructure ^b^ | - What training/resources have been most/least helpful? - What changes would you make to AI Scribe? - What would increase your use of AI Scribe? - What would encourage/support long-term sustainability of AI Scribe? |
| Organizational Characteristics ^b^ | - Alignment with health system’s goals or clinic goals - Alignment with personal goals - Receptiveness of other providers to AI scribe |
| Patient Characteristics ^b^ | - Ideal and non-ideal patients for AI Scribe |
| Reach ^c^ | - Factors that contributed to whether providers use AI scribe |
| Effectiveness ^c^ | - Whether the use of AI scribe impacts any quality and safety, clinical, or experience outcomes (to anyone or to the healthcare system, including providers, patients, families, etc) - How AI scribe did/did not impact any outcomes (i.e., mechanisms) |
| Adoption ^c^ | - Factors that contribute to providers using (or not using) AI scribe |
| Implementation ^c^ | - Comments on how AI scribe is implemented - Comments on how they have adapted/modified how they use AI Scribe to tailor it to meet their needs - Suggestions for how to better implement AI scribe (or modifications in how its design/function could be better) - Ways in which implementation of AI scribe helps/hinders any outcomes |
| Maintenance ^c^ | - Comments on amount of AI scribe use over time - What is sustained, what discontinued, what modified – why? - What would be needed to keep using AI scribe long-term |
| Trainee Curriculum ^d^ | - Comments on medical student (or resident or fellow) curriculum or use of AI scribe (now or in their future) |

AI – artificial intelligence.

^a^ Deductive code derived from the Sociotechnical Systems framework

^b^ Deductive code derived from PRISM (Practical, Robust, Implementation and Sustainability Model)

^c^ Deductive code derived from RE-AIM (Reach, Effectiveness, Adoption, Implementation, Maintenance)

^d^ Inductive code
